# TRIB1 rs17321515 gene polymorphism increases the risk of coronary heart disease in general population and non-alcoholic fatty liver disease patients in Chinese Han population

**DOI:** 10.1186/s12944-019-1108-2

**Published:** 2019-08-31

**Authors:** Qun Liu, Shou-Sheng Liu, Zhen-Zhen Zhao, Ben-Tian Zhao, Shui-Xian Du, Wen-Wen Jin, Yong-Ning Xin

**Affiliations:** 1 The Affiliated Qingdao Municipal Hospital of Qingdao University, Qingdao, 266011 China; 2 Department of Infectious Disease, The Affiliated Qingdao Municipal Hospital of Qingdao University, 1 Jiaozhou Road, Qingdao, 266011 Shandong Province China; 3 Central Laboratories, The Affiliated Qingdao Municipal Hospital of Qingdao University, Qingdao, 266071 China; 4 Department of Gastroenterology, The Affiliated Qingdao Municipal Hospital of Qingdao University, Qingdao, 266011 China; 5 Digestive Disease Key Laboratory of Qingdao, Qingdao, 266071 China

**Keywords:** Non-alcoholic fatty liver disease, TRIB1, Coronary heart disease, Polymorphism, Lipids metabolism

## Abstract

**Background:**

Present evidences suggested that *TRIB1* rs17321515 polymorphism was tightly associated with the increased risk of NAFLD and CHD. CHD is one of the main complications of NAFLD, whether *TRIB1* rs17321515 polymorphism could affect the risk of CHD in general population and NAFLD patients in Chinese Han population was remain unknown. The present study was designed to investigate the association between *TRIB1* rs17321515 polymorphism and the risk of CHD in general population and NAFLD patients in Chinese Han population, and investigate the effect of *TRIB1* rs17321515 polymorphism on serum lipid levels.

**Patients and methods:**

*TRIB1* rs17321515 gene polymorphism was genotyped using the polymerase chain reaction (PCR) in healthy controls (*n* = 175), CHD patients (*n* = 155), NAFLD patients (*n* = 146), and NAFLD+CHD patients (*n* = 156). Serum lipid profiles were determined using biochemical methods. Statistical analyses were performed using SPSS 24.0 statistical software.

**Results:**

The *TRIB1* rs17321515 AA+GA genotypes were the significant risk factors for the CHD in general population (OR = 1.788; 95% CI: 1.104–2.897; *P* = 0.018) and in the NAFLD patients (OR = 1.760; 95% CI: 1.071–2.891; *P* = 0.026). After adjusted for age, gender, and body mass index, the risk for CHD in general population (OR = 1.857; 95% CI: 1.116–3.089; *P* = 0.017) and NAFLD patients was still significant (OR = 1.723; 95% CI: 1.033–2.873; *P* = 0.037). In addition, *TRIB1* rs17321515 A carriers possess the higher lipid profiles in the included subjects.

**Conclusions:**

*TRIB1* rs17321515 AA+GA genotypes were significant associated with the risk of CHD in general population and in NAFLD patients in Chinese Han population. The rs17321515 A allele increases the serum lipid profiles in included subjects.

## Background

Non-alcoholic fatty liver disease (NAFLD) is defined histologically as when more than 5% of liver cells are found to be suffering from steatosis, which is the manifestation of metabolic syndrome in liver [1]. The disease spectrum of NAFLD includes non-alcoholic fatty liver (NAFL), non-alcoholic steatohepatitis (NASH), fibrosis, liver cirrhosis and even hepatocellular carcinoma [2]. NAFLD has exceeded viral hepatitis and become the most prevalent liver disease in the world, and become the second largest cause of liver transplantation and the third largest cause of liver cancer [3]. Coronary heart disease (CHD) is a form of the metabolic syndrome, more and more epidemiology and clinical studies had been conducted to investigate the tightly association of NAFLD and CHD [4]. Increased evidences suggested that NAFLD is not only a marker of CHD and other structural, functional and arrhythmia complications, but also takes effects in the development and progression of these cardiac complications [5, 6]. Therefore, patients with NAFLD will benefited from the more rigorous monitoring and early treatment interventions to reduce the risk of CHD and other cardiac and arrhythmia complications.

NAFLD and CHD possess several the same influence factors such as lipids metabolism disorder, obesity and insulin resistance [7]. In addition, genetic factor such as gene polymorphism was also the significant risk factor for the NAFLD and CHD [8–11]. Genome-wide association study (GWAS) revealed many gene polymorphisms such as *PNPLA3* rs738409 and *TM6SF2* rs58542926 which were the significant risk factors for NAFLD [12, 13]. Abundant studies revealed that gene polymorphisms such as *CD36* rs1761667, *CDKN2BAS* rs496892, and *COX2* -765G > C were tightly associated with the risk of CHD in different countries [14–16]. Tribbles 1 (*TRIB1*) encoding Tribbles protein homolog 1 [17], proteins from the tribbles family include a MEK1-binding domain, an E3 ubiquitin ligase (COP1)-binding domain, and a pseudokinase domain, all of which are significant by interacting with respective partners [18]. GWAS in American identified that lots of novel genomic loci participated in the regulation of plasma triglyceride (TG) levels. One of those loci was the 8q24 locus, with the lead single nucleotide polymorphism (SNP) involving a linkage-disequilibrium block that contains the gene TRIB1 [19]. Although TRIB1 undergoes miRNA regulation, it is quite conserved in parts, containing a long 1.5 Kbp 3′ untranslated region (UTR) [20]. Under normal physiological conditions, TRIB1 can negatively regulate carbohydrate-responsive element-binding protein (ChREBP) via proteasome proteolysis ubiquitination, raising the microsomal triglyceride transfer protein (MTTP) to terminate lipogenesis. In the absence of TRIB1, the process of lipogenesis is aberrant and the serum lipid disorders was occurred [21]. GWAS studies have repeatedly confirmed that TRIB1 gene polymorphisms were associated with dyslipidemia [22–25], and several studies had shown that TRIB1 gene variants can increase the risk of CHD [26, 27]. In addition, previous studies have confirmed that *TRIB1* gene variation can also increase the risk of NAFLD [28–30].

In consideration of the prevalent complication of NAFLD and CHD, understanding of effect of genetic factors on the patients with NAFLD and CHD were urgent and remains to be explored. The aim of this study was to investigate the association of *TRIB1* rs17321515 gene polymorphism with the risk of CHD in NAFLD patients in Chinese Han population, and investigate the effect of *TRIB1* rs17321515 on the serum lipid profiles of patients with NAFLD and CHD.

## Patients and methods

### Study subjects

This study was approved by the Ethical Committee of Qingdao Municipal Hospital (Qingdao, China), and this study was conducted in accordance with the principles of the Helsinki declaration and its appendices [31]. All the subjects had signed the informed consent before participating in this study.

From June 2018 to November 2018, 146 NAFLD patients (65 females and 81 males, median age 64.00 years) diagnosed by B-type ultrasonography, 155 CHD patients (72 females and 83 males, median age 60.00 years) diagnosed by coronary angiography, 156 patients (80 females and 76 males, median 64.00 years) with both NAFLD and CHD diagnosed by B-type ultrasonography and coronary angiography, and 175 healthy controls (87 females and 88 males, median 62.00 years) that matched for sex and age were enrolled in this study. Clinical data of all the subjects were collected from the department of Gastroenterology and Cardiology, and the Medical center of Qingdao Municipal Hospital. All the individuals were unrelated Northern Han Chinese origin. NAFLD was diagnosed by a standard clinical evaluation, according to the criteria of the American association for the study of liver diseases (AASLD) [32]. CHD was diagnosed by a percutaneous coronary angiography, with the presence of at least 50% stenosis in at least one of the coronary arteries. Subjects who had other liver diseases, other cardiac disorders or diabetes mellitus were excluded.

### Biochemical analyses

The basic clinical information (name, gender, age, body height, and weight) were obtained by a standard study questionnaire. The body mass index (BMI) was calculated using the equation that mass (kg)/height (m)^2^. After a 12-h overnight fasting, blood sample was collected from median vein of each subject, and the blood sample was placed into an ethylene diamine tetraacetic acid (EDTA)-containing tube. The serum levels of alanine aminotransferase (ALT), aspartate aminotransferase (AST), gamma-glutamyltranspeptidase (GGT), alkaline phosphatase (ALP), triglyceride (TG), total cholesterol (TC), high-density lipoprotein (HDL), low-density lipoprotein (LDL), total bilirubin (TBIL), fasting plasma glucose (FPG) were measured by standard clinical laboratory techniques (IChem-540 automatic biochemical analyzer, Shenzhen, China), respectively. Environmental factors were excluded in the study.

### Genomic DNA extraction and genotyping

Blood genomic DNA was isolated with a genomic DNA purification kit (TIANGEN, Beijing, China) according to the manufacturer’s instructions and stored at − 20 °C until use. The genotyping of TRIB1 rs17321515 was conducted using the polymerase chain reaction (PCR). Primers for PCR were 5′-ACGTTGGATGTAGAAGTCCCCTTCCCTTAG-3′ and 5′-ACGTTGGATGGAACAAGGACTTTCGTCCTC-3′. PCR amplification was performed under the following conditions: an initial denaturation at 94 °C for 5 min, followed by 45 cycles of denaturing at 94 °C for 20 s, annealing at 56 °C for 30 s, and extending at 72 °C for 1 min. The genotypes of rs17321515 were detected by direct DNA sequencing using the ABI veriti-384 Prism sequence detection system, and the raw data were analyzed using MassARRAY TYPER4.0 software (Agena, Inc). Genotyping was performed in blinded fashion and the success rates were > 95%.

### Statistical analysis

Statistical analysis was performed using the statistical package for the social sciences (SPSS) version 24.0 (SPSS Inc. Chicago, IL, USA). The Hardy-Weinberg equilibrium of expected and observed genotype distribution was analyzed by Pearson’s χ^2^ test. After testing for normality, continuous variables were shown as the mean ± standard deviation or median (interquartile range) for normal and abnormal distributed parameters, respectively. The t-test and Wilcoxon rank sum test were used for comparison of continuous data between groups. The Genotypes and allele frequencies were evaluated using the χ^2^ test. The association between SNP and CHD risk in NAFLD patients was estimated by computing odds ratios (ORs) and 95% confidence intervals (CI) from the binary logistic regression analyses. *P* < 0.05 was considered as statistically significant.

## Results

### Clinical characteristics of the study participants

**Fig. 1:**
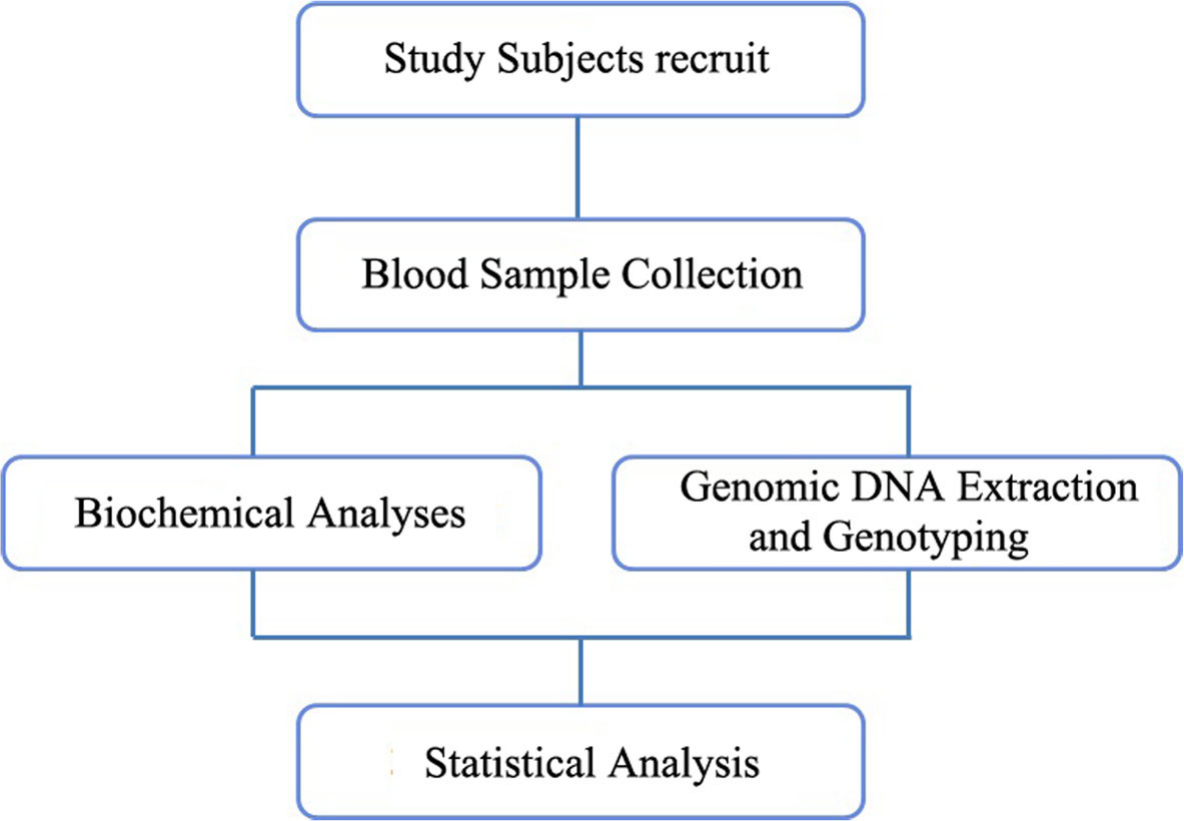
The flow chart of this study

The flow chart of this study was shown in the Fig. 1. The clinical characteristics of CHD patients and healthy controls are shown in the Table 1. The two groups were matched for gender and age (all *P* > 0.05). The CHD patients had higher BMI value and serum levels of ALT, GGT, TG and FPG than healthy controls (all *P* < 0.05), besides, the serum level of HDL and LDL in CHD patients was significant lower compared to the healthy controls (all *P* < 0.05). The clinical characteristics of NAFLD+CHD patients and NAFLD patients are shown in the Table 2. The NAFLD+CHD patients had higher serum levels of ALP, TBIL, FPG than NAFLD patients (all *P* < 0.05), lower BMI and HDL than NAFLD patients (all *P* < 0.05).

**Table 1:** Clinical Characteristics of CHD Patients and Healthy controls

Parameters	CHD (*n* = 155)	Controls (*n* = 175)	Statistics	*P* value
Male/Female	83/72	88/87	χ^2^ = 0.350	0.554
Age, y	60.00 (59.00, 75.00)	62.00 (53.00, 68.00)	Z = −1.013	0.311
BMI, kg/m^2^	24.20 ± 2.73	22.44 ± 2.88	t = 5.632	< 0.001
ALT, U/L	19.21 (14.57, 33.40)	17.96 (12.57, 24.20)	Z = −2.239	0.025
AST, U/L	22.45 (16.70, 37.78)	20.78 (17.60, 24.35)	Z = −1.924	0.054
GGT, U/L	23.75 (17.09, 36.74)	19.91 (15.24, 29.92)	Z = −2.117	0.034
ALP, U/L	83.84 (66.72, 109.08)	69.71 (57.27, 83.63)	Z = −1.083	0.279
TG, mmol/L	1.23 (0.90, 1.61)	1.12 (0.86, 1.62)	Z = −3.026	0.002
TC, mmol/L	4.19 (3.71, 5.13)	4.64 (3.66, 5.18)	Z = −1.525	0.127
HDL, mmol/L	1.00 (0.85, 1.20)	1.31 (1.05, 1.47)	Z = −7.493	< 0.001
LDL, mmol/L	2.49 (2.06, 3.18)	3.01 (2.61, 3.56)	Z = −3.552	< 0.001
TBIL, μmol/L	13.50 (10.60, 17.96)	12.10 (9.50, 15.10)	Z = − 0.366	0.714
FPG, mmol/L	5.02 (4.49, 6.02)	4.54 (4.06, 5.05)	Z = −5.879	< 0.001
pharmacological history	lipid-lowering drug	/		

*Abbreviations*: *ALP*, alkaline phosphatase; *ALT*, alanine aminotransferase; *AST*, aspartate aminotransferase; *BMI*, body mass index; *FPG*, fasting plasma glucose; *GGT*, γ-glutamyltransferase; *HDL*, high-density lipoprotein; *LDL*, low-density lipoprotein; *TBIL*, total bilirubin; *TC*, total cholesterol; *TG*, triglyceride

**Table 2:** Clinical Characteristics of NAFLD+CHD Patients and NAFLD Patients

Parameters	NAFLD+CHD (*n* = 156)	NAFLD (*n* = 146)	Statistics	*P* value
Male/Female	76/80	81/65	χ^2^ = 0.421	0.516
Age, y	64.00 (59.00, 72.75)	64.00 (45.00, 74.00)	Z = − 0.644	0.520
BMI, kg/m^2^	25.47 (23.05, 27.68)	26.35 (24.86, 28.49)	Z = −3.431	0.001
ALT, U/L	23.58 (16.07, 34.36)	22.67 (18.30, 35.68)	Z = − 0.919	0.358
AST, U/L	21.29 (16.80, 32.02)	21.55 (18.38, 28.09)	Z = − 0.647	0.517
GGT, U/L	30.70 (21.31, 42.67)	32.55 (20.97, 47.79)	Z = − 0.986	0.324
ALP, U/L	79.52 (66.58, 96.46)	68.48 (59.09, 81.81)	Z = −3.124	0.002
TG, mmol/L	1.58 (1.13, 2.18)	1.54 (1.16, 2.21)	Z = −0.145	0.885
TC, mmol/L	4.64 (3.75, 5.54)	5.42 (4.98, 6.02)	Z = −1.682	0.093
HDL, mmol/L	1.02 (0.88, 1.12)	1.19 (1.05, 1.32)	Z = −6.741	< 0.001
LDL, mmol/L	2.86 (2.10, 3.45)	3.30 (2.97, 3.52)	Z = − 1.332	0.183
TBIL, μmol/L	12.70 (9.70, 16.77)	12.00 (10.00, 14.90)	Z = − 0.842	0.040
FPG, mmol/L	5.70 (4.68, 6.96)	4.96 (4.52, 5.79)	Z = −3.503	< 0.001

*Abbreviations*: *ALP*, alkaline phosphatase; *ALT*, alanine aminotransferase; *AST*, aspartate aminotransferase; *BMI*, body mass index; *FPG*, fasting plasma glucose; *GGT*, γ-glutamyltransferase; *HDL*, high-density lipoprotein; *LDL*, low-density lipoprotein; *TBIL*, total bilirubin; *TC*, total cholesterol; *TG*, triglyceride

### Genotype and allele distributions of TRIB1 rs17321515

The genotype distributions of *TRIB1* rs17321515 in the four groups were in accordance with the Hardy-Weinberg equilibrium (all *P* > 0.05) (Table 3). As described in the Table 4, there was significant difference in the allele distribution of *TRIB1* rs17321515 between CHD patients and controls group (*P* = 0.031). The *TRIB1* rs17321515 GA + AA genotypes were the significant risk factor for the development of CHD (OR = 1.788; 95% CI: 1.104–2.897; *P* = 0.018), after adjusted for age, gender, and body mass index, the risk of *TRIB1* rs17321515 GA + AA genotypes were still marked (OR = 1.857; 95% CI: 1.116–3.089; *P* = 0.017) (Table 5). In addition, there was significant difference in the allele distribution of *TRIB1* rs17321515 between NAFLD+CHD patients and NAFLD group (*P* = 0.021) (Table 4). The *TRIB1* rs17321515 GA + AA genotypes were the significant risk factor for the development of CHD in NAFLD patients (OR = 1.760; 95% CI: 1.071–2.891; *P* = 0.026), after adjusted for age, gender, and body mass index, the risk of *TRIB1* rs17321515 GA + AA genotypes were still marked (OR = 1.723; 95% CI: 1.033–2.873; *P* = 0.037) (Table 5).

**Table 3:** Results of the Hardy-Weinberg Equilibrium

	χ^2^	*P* value
Control	1.693	0.193
CHD	0.091	0.762
NAFLD+CHD	0.003	0.957
NAFLD	0.403	0.526

**Table 4:** Distributions of the TRIB1 rs17321515 Genotypes and Alleles in the Study Groups

	NAFLD	CHD	NAFLD+CHD	Control	χ2	*P* 1	χ2	*P* 2
Genotypes					5.668	0.059	5.646	0.059
GG	37 (26.6)	39 (22.8)	60 (39.0)	67 (40.6)				
AG	73 (52.5)	72 (42.1)	72 (46.8)	70 (42.4)				
AA	29 (20.9)	30 (35.1)	22 (14.3)	28 (17.0)				
Alleles					4.641	0.031	5.363	0.021
G	147 (52.9)	150 (53.2)	192 (62.3)	204 (61.8)				
A	131 (47.1)	132 (46.8)	116 (37.7)	126 (38.2)				

*P* 1:CHD vs Control; *P* 2:NAFLD+CHD vs NAFLD

**Table 5:** Odds Ratios According to Genotypes of TRIB1 rs17321515 Gene Polymorphism in Study Group

	Unadjusted				Adjusted^a^			
	OR (95% CI)	*P*1	OR (95% CI)	*P*2	OR (95% CI)	*P*1	OR (95% CI)	*P*2
GG	1		1		1		1	
GA + AA	1.788 (1.104–2.897)	0.018	1.760 (1.071–2.891)	0.026	1.857 (1.116–3.089)	0.017	1.723 (1.033–2.873)	0.037

^a^ Binary logistic regression model was adjusted for age, gender, and body mass index

*P* 1: CHD vs Control; *P* 2: NAFLD+CHD vs NAFLD

### Association of the TRIB1 rs17321515 gene polymorphism with clinical parameters characteristics in each group subjects

We compared the *TRIB1* rs17321515 A allele with the clinical characteristics of the four groups to estimate whether the rs17321515 A allele was correlated with clinical parameters. As the results shown in the Table 6, there was no significant difference between the A allele carriers and non-carriers in the overall series and in the NAFLD patients (all *P* > 0.05) (Tables 6 and 7). Higher serum level of FPG in the A allele carriers was observed compared to the non-carriers in the CHD group (*P* = 0.021) (Table 8). In the NAFLD+CHD patients, the A allele carriers had the higher serum HDL level than non-carriers (*P* = 0.015) (Table 9). In the healthy controls, the A allele carriers had the higher serum TC level than non-carriers (*P* = 0.001) (Table 10).

**Table 6:** Clinical Characteristics of TRIB1 rs17321515 A allele Carriers and Non-carriers in the Overall Series

Parameters	Carriers (*n* = 396)	Non-carriers (*n* = 203)	Statistics	*P* value
Male/Female	206/190	104/99	χ^2^ = 0.033	0.855
Age, y	64.00 (54.25, 72.00)	65.00 (54.00, 74.00)	Z = −1.221	0.222
BMI, kg/m^2^	24.66 (22.51, 26.83)	24.73 (22.39, 26.47)	Z = −0.563	0.573
ALT, U/L	21.31 (15.50, 32.31)	20.88 (14.42, 29.42)	Z = − 1.275	0.202
AST, U/L	21.32 (17.63, 29.36)	21.13 (17.15, 26.80)	Z = − 0.804	0.422
GGT, U/L	26.64 (18.73, 40.29)	25.32 (16.85, 39.86)	Z = −1.577	0.115
ALP, U/L	75.65 (62.23, 91.77)	73.06 (59.43, 87.69)	Z = −1.691	0.091
TG, mmol/L	1.41 (0.98, 1.96)	1.31 (0.97, 1.83)	Z = −1.427	0.153
TC, mmol/L	4.72 (3.89, 5.54)	4.86 (4.03, 5.41)	Z = − 0.603	0.546
HDL, mmol/L	1.10 (0.95, 1.31)	1.08 (0.93, 1.32)	Z = − 0.577	0.564
LDL, mmol/L	3.00 (2.36, 3.47)	2.99 (2.48, 3.44)	Z = −0.207	0.836
TBIL, umol/L	12.70 (9.80, 16.27)	13.20 (10.00, 17.00)	Z = − 0.692	0.489
FPG, mmol/L	4.95 (4.41, 5.77)	4.96 (4,41, 6.11)	Z = −0.730	0.466

**Table 7:** Clinical Characteristics of TRIB1 rs17321515 A allele Carriers and Non-carriers in the NAFLD Patients

Parameters	Carriers (*n* = 102)	Non-carriers (*n* = 37)	Statistics	*P* value
Male/Female	54/48	20/17	χ^2^ = 0.014	0.907
Age,y	64.00 (45.00, 74.00)	67.00 (45.00, 75.50)	Z = −0.851	0.395
BMI, kg/m^2^	26.47 (24.87, 28.74)	26.23 (24.62, 28.06)	Z = −0.110	0.913
ALT, U/L	22.84 (18.27, 36.72)	22.34 (17.79, 36.40)	Z = −0.434	0.665
AST, U/L	21.55 (18.84, 28.10)	21.55 (17.33, 27.56)	Z = −0.591	0.555
GGT, U/L	33.15 (22.38, 47.95)	29.14 (18.47, 47.54)	Z = −1.075	0.285
ALP, U/L	71.83 ± 15.31	68.47 ± 19.99	t = 1.049	0.296
TG, mmol/L	1.73 (1.16, 2.26)	1.44 (1.17, 2.17)	Z = −0.624	0.532
TC, mmol/L	5.42 ± 0.80	5.53 ± 0.87	t = − 0.688	0.493
HDL, mmol/L	1.18 (1.05, 1.29)	1.21 (1.02, 1.35)	Z = − 0.331	0.740
LDL, mmol/L	3.30 (2.95, 3.50)	3.40 (3.17, 3.67)	Z = − 1.833	0.067
TBIL, umol/L	11.90 (9.80, 14.62)	13.90 (10.10, 17.90)	Z = − 1.258	0.208
FPG, mmol/L	4.94 (4.50, 5.80)	4.96 (4.61, 5.96)	Z = − 0.060	0.952

**Table 8:** Clinical Characteristics of TRIB1 rs17321515 A allele Carriers and Non-carriers in the CHD Patients

Parameters	Carriers (*n* = 102)	Non-carriers (*n* = 39)	Statistics	*P* value
Male/Female	50/52	20/19	χ^2^ = 0.058	0.810
Age, y	66.12 ± 9.96	68.10 ± 12.61	t = − 0.883	0.381
BMI, kg/m^2^	24.22 (22.76, 25.71)	24.51 (22.50, 26.26)	Z = − 0.199	0.843
ALT, U/L	18.88 (14.74, 33.03)	20.27 (12.81, 43.41)	Z = −0.018	0.985
AST, U/L	22.13 (16.22, 36.05)	22.73 (16.89, 43.23)	Z = −0.249	0.803
GGT, U/L	23.64 (17.30, 36.65)	25.98 (16.89, 46.81)	Z = −0.350	0.726
ALP, U/L	84.87 (67.18, 110.11)	74.64 (62.40, 108.33)	Z = − 0.843	0.399
TG, mmol/L	1.21 (0.90, 1.69)	1.27 (0.92, 1.59)	Z = − 0.549	0.583
TC, mmol/L	4.15 (3.59, 5.11)	4.07 (3.68, 5.13)	Z = − 0.108	0.914
HDL, mmol/L	0.98 (0.85, 1.16)	1.01 (0.82, 1.25)	Z = − 0.904	0.366
LDL, mmol/L	2.49 (2.02, 3.23)	2.30 (2.08, 3.17)	Z = − 0.452	0.651
TBIL, umol/L	13.30 (10.67, 17.92)	15.40 (10.60, 18.69)	Z = − 0.924	0.355
FPG, mmol/L	5.31 (4.82, 6.43)	4.89 (4.37, 5.55)	Z = −2.307	0.021

**Table 9:** Clinical Characteristics of TRIB1 rs17321515 A allele Carriers and Non-carriers in the NAFLD+CHD Patients

Parameters	Carriers (*n* = 94)	Non-carriers (*n* = 60)	Statistics	*P* value
Male/Female	50/44	30/30	χ^2^ = 0.149	0.699
Age, y	63.34 ± 10.53	66.58 ± 10.92	t = −1.837	0.068
BMI, kg/m^2^	25.54 ± 3.24	25.35 ± 3.21	t = 0.344	0.731
ALT, U/L	23.72 (17.16, 37.60)	23.30 (14.58, 32.86)	Z = − 1.106	0.269
AST, U/L	21.72 (17.37, 32.15)	20.12 (15.67, 32.54)	Z = −0.728	0.467
GGT, U/L	32.39 (22.76, 44.39)	29.28 (19.67, 40.77)	Z = −1.384	0.166
ALP, U/L	80.61 (65.89, 98.21)	76.27 (62.27, 89.86)	Z = −0.947	0.344
TG, mmol/L	1.70 (1.31, 2.21)	1.40 (1.00, 2.09)	Z = −1.932	0.053
TC, mmol/L	4.66 (3.73, 5.67)	4.54 (3.75, 5.34)	Z = −0.943	0.346
HDL, mmol/L	1.04 (0.92, 1.17)	0.95 (0.82, 1.08)	Z = −2.435	0.015
LDL, mmol/L	2.80 (2.08, 3.46)	2.88 (2.03, 3.33)	Z = −0.369	0.712
TBIL, umol/L	13.30 (9.70, 17.07)	12.48 (9.42, 16.48)	Z = −0.513	0.608
FPG, mmol/L	5.64 (4.65, 7.16)	5.79 (4.67, 6.87)	Z = −0.371	0.711

**Table 10:** Clinical Characteristics of TRIB1 rs17321515 A allele Carriers and Non-carriers in the Healthy Controls

Parameters	Carriers (*n* = 98)	Non-carriers (*n* = 67)	Statistics	*P* value
Male/Female	52/46	34/33	χ^2^ = 0.085	0.770
Age, y	62.00 (53.00, 69.00)	63.00 (52.00, 68.00)	Z = −0.349	0.727
BMI, kg/m^2^	22.27 ± 2.81	22.66 ± 3.02	t = − 0.850	0.397
ALT, U/L	17.93 (12.50, 25.86)	19.70 (12.57, 23.82)	Z = −0.041	0.967
AST, U/L	19.94 (12.28, 25.69)	20.87 (18.10, 23.01)	Z = −0.075	0.940
GGT, U/L	20.05 (15.15, 30.21)	19.59 (15.59, 31.89)	Z = −0.071	0.943
ALP, U/L	69.92 (57.31, 82.96)	66.73 (54.60, 83.63)	Z = −0.493	0.622
TG, mmol/L	1.13 (0.86, 1.65)	1.08 (0.88, 1.67)	Z = −0.017	0.987
TC, mmol/L	4.85 (4.30, 5.32)	4.24 (3.44, 5.00)	Z = −3.263	0.001
HDL, mmol/L	1.35 (1.06, 1.51)	1.28 (1.03, 1.40)	Z = −1.175	0.240
LDL, mmol/L	3.11 ± 0.77	3.09 ± 0.59	t = 0.173	0.863
TBIL, umol/L	11.55 (9.37, 15.22)	12.30 (9.50, 15.10)	Z = −0.484	0.628
FPG, mmol/L	4.56 (4.11, 5.05)	4.50 (4.04, 4.97)	Z = −0.616	0.538

## Discussion

NAFLD has become a worldwide public health problem. Clinical experience and epidemiological evidences showed that NAFLD-related mortality not only referred to the liver itself, but also companied with the complication such as the increased risk of CHD [33, 34]. NAFLD could aggravate insulin resistance in the body and liver that cause atherosclerotic dyslipidemia, and release a variety of pro-inflammatory, pro-coagulant, and pro-fibrotic mediators, which may play an important role in the pathophysiology of cardiac and arrhythmia complications [33, 35]. Single nucleotide polymorphism (SNP) as a significant genetic factor, plays an important role in the development of NAFLD and CHD [36, 37]. Multiple studies have confirmed that *TRIB1* gene polymorphisms can increase the risk of NAFLD and the risk of CHD [28, 29, 38]. In this study, we investigated the relationship of *TRIB1* rs17321515 gene polymorphism with the risk of CHD, and the risk of CHD in NAFLD patients in Chinese Han population for the first time. The results showed that *TRIB1* rs17321515 GA + AA genotypes were significantly associated with the increased risk of CHD in healthy controls and NAFLD patients.

Tribbles-1 (TRIB1) is one of the member of tribbles family which were first identified in *Drosophila* and possess the function to regulate the cell division and migration [39]. Previous studies confirmed that *TRIB1* encodes a human homologue of the Drosophila tribbles protein, and the relationship of *TRIB1* variant with the serum lipids metabolism was discovered by GWAS in the European population [40, 41]. To further explore the role of TRIB1 in lipoprotein metabolism, Burkhardt et al. found the serum levels of TG and cholesterol were inversely associated with the expression of hepatic TRIB1 in mice [17]. Several studies had reported the effect of *TRIB1* rs17321515 on the serum lipids metabolism. In a Chinese Han cohort study, *TRIB1* rs17321515 AA genotype carriers had higher TG level than GG genotype carriers [42]. Katalin et al. also found that *TRIB1* rs17321515 AA genotype carriers had higher serum TG and TC levels than GG + GA genotypes carriers [43]. In consideration of the higher mortality rate of NAFLD-related CHD than the single liver disease, it is meaningful to investigate the effect of *TRIB1* rs17321515 gene polymorphism on the risk of CHD in general population and NAFLD patients, and the effect on the serum lipids levels [4, 44]. In this study, we observed that *TRIB1* rs17321515 AA+GA genotypes were significantly associated with the risk of CHD in general Chinese Han population, Besides, *TRIB1* rs17321515 AA+GA genotypes carriers in NAFLD patients had a higher CHD risk than GG genotype carriers. Our results showed that *TRIB1* rs17321515 polymorphism was a significant risk factor for CHD in the general population and could increase the CHD risk in NAFLD patients in Chinese Han population. Moreover, *TRIB1* rs17321515 A allele could affect the serum lipids levels such as FPG, HDL, and TC in each group. Our conclusion is consistent with the previous studies which were conducted in other countries [26, 43, 45].

Some limitations of our study must be acknowledged. Firstly, all the included subjects in this study are Chinese Han nationality, our conclusion may not be applicable to other nationality absolutely. Secondly, lacking of liver biopsy is the main limitation of our study, however, liver biopsy is invasive, and may lead to a small probability of serious morbidity. Thereby, we used abdominal ultrasound to diagnose NAFLD in the present study. Thirdly, some of our subjects were long-term users of lipid-lowering drugs, which will affect the accuracy of the results to some extent. Lastly, nutraceuticals can influence the lipids levels [46], but we did not consider this factor in this study.

## Conclusion

In summary, we investigated the relationship of *TRIB1* rs17321515 with the risk of CHD in general population and NAFLD patients in Chinese Han population for the first time. Our results showed that *TRIB1* rs17321515 AA+GA genotypes are associated with an increased risk of CHD in general population and NAFLD patients, and the rs17321515 A allele affects the serum lipids levels of multiple lipid profiles in included subjects. Diverse ethnic groups and larger sample sizes are needed to investigate in further study to confirm the present data.

## Data Availability

None.

## Abbreviations

AASLD: American association for the study of liver diseases
ALP: Alkaline phosphatase
ALT: Alanine aminotransferase
AST: Aspartate aminotransferase
BMI: Body mass index
CHD: Coronary heart disease
ChREBP: Carbohydrate-responsive element-binding protein;
CI: Confidence intervals
EDTA: Ethylene diamine tetraacetic acid
FPG: Fasting plasma glucose
GGT: Gamma-glutamyltranspeptidase
GWAS: Genome-wide association study
HDL: High density lipoprotein
LDL: Low density lipoprotein
MTTP: Microsomal triglyceride transfer protein
NAFL: Non-alcoholic fatty liver
NAFLD: Non-alcoholic fatty liver disease
NASH: Non-alcoholic steatohepatitis
ORs: Odds ratios
PCR: Polymerase chain reaction
SNP: Single nucleotide polymorphism
TBIL: Total bilirubin
TC: Total cholesterol
TG: Triglycerides
TRIB1: Tribbles 1
